# Heterogeneous GAD65 Expression in Subtypes of GABAergic Neurons Across Layers of the Cerebral Cortex and Hippocampus

**DOI:** 10.3389/fnbeh.2021.750869

**Published:** 2021-11-03

**Authors:** Yuki Kajita, Hajime Mushiake

**Affiliations:** Department of Physiology, Graduate School of Medicine, Tohoku University, Sendai, Japan

**Keywords:** cerebral cortex, GABAergic subtype, GAD65, hippocampus, rat

## Abstract

Gamma-aminobutyric acid (GABA), a major inhibitory transmitter in the central nervous system, is synthesized via either of two enzyme isoforms, GAD65 or GAD67. GAD65 is synthesized in the soma but functions at synaptic terminals in an activity-dependent manner, playing a distinct role in excitatory-inhibitory balance. However, the extent to which each GABAergic subtype expresses GAD65 in the resting state remains unclear. In this study, we compared GAD65 expression among six GABAergic subtypes: NPY^+^, nNOS^+^, PV^+^, SOM^+^, CR^+^, and CCK^+^. According to the results, the GABAergic subtypes were classified into two groups per region based on GAD65 expression levels: high-expression (NPY^+^ and nNOS^+^) and low-expression groups (PV^+^, SOM^+^, CR^+^, and CCK^+^) in the cerebral cortex and high-expression (NPY^+^, nNOS^+^, and CCK^+^) and low-expression groups (PV^+^, SOM^+^, and CR^+^) in the hippocampus. Moreover, these expression patterns revealed a distinct laminar distribution in the cerebral cortex and hippocampus. To investigate the extent of GAD65 transport from the soma to synaptic terminals, we examined GAD65 expression in colchicine-treated rats in which GAD65 was synthesized in the soma but not transported to terminals. We found a significant positive correlation in GAD65 expression across subtypes between colchicine-treated and control rats. In summary, each GABAergic subtype exhibits a distinct GAD65 expression pattern across layers of the cerebral cortex and hippocampus. In addition, the level of GAD65 expression in the soma can be used as a proxy for the amount of GAD65 in the cytoplasm. These findings suggest that exploration of the distinct profiles of GAD65 expression among GABAergic subtypes could clarify the roles that GABAergic subtypes play in maintaining the excitatory-inhibitory balance.

## Introduction

The inhibitory neurotransmitter gamma-aminobutyric acid (GABA) is widely distributed in the central nervous system and is synthesized from glutamate via two isoforms of glutamic acid decarboxylase (GAD): GAD67 and GAD65. These two isoforms are encoded by distinct genes but are co-expressed in most GABAergic interneurons (Erlander et al., 1991; Feldblum et al., 1993). The mRNA (Dicken et al., 2015) and protein (Rimvall et al., 1993) expression of GAD67 is closely related to GABA content, as GAD67 is responsible for basal and tonic GABA synthesis (Asada et al., 1997; Chattopadhyaya et al., 2007; Obata et al., 2008). On the other hand, GAD65 has a high affinity for the cofactor pyridoxal 5′-phosphate (Martin and Rimvall, 1993), and localizes strongly in axon terminals rather than in the soma (Esclapez et al., 1994). In addition, repeated electrical stimulation has been shown to increase GABA contents and its release (Bowdler et al., 1983) while increasing GAD65 expression (Jinno and Kosaka, 2009). These previous data suggest that GAD65 expression is closely related to activity-dependent and phasic GABA synthesis.

GAD67 and GAD65 expression are not evenly distributed within GABAergic neurons in various brain regions, including the cerebral cortex and hippocampus (Esclapez et al., 1994; Hendrickson et al., 1994; Houser and Esclapez, 1994). In particular, GAD65 expression in the soma is heterogeneous compared with that of GAD67 (Guo et al., 1997). Although the drivers of GAD65 expression remain unknown, this heterogeneous expression may depend on the diversity of GABAergic neuron properties. One previous study showed that GAD65 expression is lower in calcium-binding protein parvalbumin-positive (PV^+^) cells in the hippocampus (Fukuda et al., 1997), suggesting that GAD65 expression depends on the interneuron subtype. GABAergic neurons are classified into multiple subtypes in addition to PV^+^ cells based on chemical markers (Hendry et al., 1984; Gonchar and Burkhalter, 1997; Kubota and Kawaguchi, 1997; Jinno et al., 2001; Kawaguchi and Kondo, 2002; Gonchar et al., 2007). Elucidating the relationship between GABAergic subtypes and GAD65 expression would clarify the roles of GABAergic subtypes in activity-dependent and phasic inhibition under pathological conditions such as epileptic seizures and mental disorders.

To achieve this purpose, we compared GAD65 expression among several subtypes of neurons in the cerebral cortex and hippocampus under normal conditions. Although a variety of markers can be used to identify GABAergic subtypes, we followed the classification described in previous reports (Druga, 2009; Tricoire et al., 2010; Rudy et al., 2011; Tricoire and Vitalis, 2012; Perrenoud et al., 2013; Tremblay et al., 2016) and analyzed the following six subtypes: PV^+^ neurons, somatostatin-positive (SOM^+^) neurons, calretinin-positive (CR^+^) neurons, cholecystokinin-positive (CCK^+^) neurons, neuropeptide Y-positive (NPY^+^) neurons, and neuronal nitric oxide synthase-positive (nNOS^+^) neurons. This classification system was shared between the cerebral cortex and hippocampus. We also compared the laminar distribution of GAD65 expression by dividing the cerebral cortex and hippocampus into three layers: the superficial layer, pyramidal layer and deep layer. Additionally, to confirm that somatic expression reflects expression throughout the cytoplasm, we performed the same experiments using colchicine, which blocks axonal transport.

Briefly, each GABAergic subtype exhibited a distinct GAD65 expression pattern across the cerebral cortical and hippocampal layers. Additionally, GAD65 expression in the soma of different subtypes was significantly and positively correlated between colchicine-treated and control rats.

## Materials and Methods

### Animals

We used male 8–12 weeks old Long-Evans rats (Institute for Animal Reproduction, Kasumigaura, Ibaraki, Japan, http://www.iar.or.jp) weighing 300–500 *g* each. These rats were housed in groups in a temperature- and humidity-controlled room under a 12-h-light/12-h-dark cycle with food and water available *ad libitum*. Efforts were made to minimize the number of animals used, as well as their pain and discomfort. All animal treatments were approved by the Tohoku University Committee for Animal Research, Seiryo Campus (Sendai, Japan).

### Intraventricular Injection of Colchicine

To block axonal transport, colchicine (Sigma-Aldrich, Tokyo, Japan) dissolved in saline (100 μg/10 μl) was stereotactically injected 5 μl of solution into the bilateral lateral ventricle of the brain. Control group was injected equal (5 μl) volume of saline into the bilateral lateral ventricle of the brain. The surgery was performed under inhalation anesthesia with isoflurane (1–3%, Pfizer, Tokyo, Japan). 2 days later, the brains were processed for immunohistochemical investigation, as described below.

### Tissue Preparation

Under deep nembutal anesthesia (Somnopentyl, 250 mg/kg body weight, intraperitoneally, Kyoritsu Seiyaku Co., Ltd., Tokyo Japan), the rats were transcardially perfused with saline containing 2 IU/mL heparin and then with 4% paraformaldehyde in 0.1M phosphate buffer (pH 7.4). The brains were removed and post-fixed in formaldehyde solution for 24 h at 4°C. Then, the brains were immersed in 30% sucrose solution in phosphate-buffered saline (PBS) for at least 24 h, and coronal sections (30 μm thick) were cut using a cryostat (Leica CM1950, Leica Microsystems, Nussloch, Germany).

### Immunohistochemistry

The sections were washed in PBS for 10 min. After blocking with 3% bovine serum albumin in PBS for 1 h, the sections were incubated with primary antibodies overnight at 4°C and washed with PBS. The following antibodies were used in this study: mouse monoclonal anti-GAD65 antibody (1:300, BD Biosciences, San Jose, CA, United States), rabbit polyclonal anti-parvalbumin antibody (1:500, Abcam, Cambridge, United Kingdom), rabbit polyclonal anti-somatostatin antibody (1:500; Peninsula Lab, San Carlos, CA, United States), rabbit polyclonal anti-calretinin antibody (1:500, Proteintech group, Inc., Chicago, IL, United States), rabbit polyclonal anti-cholecystokinin antibody (1:100, Cloud-Clone Corp, Carlsbad, CA, United States), rabbit polyclonal anti-neuropeptide Y antibody (1:100, Proteintech group, Inc.), rabbit polyclonal anti-neuronal nitric oxide synthase antibody (1:800, Merck-Millipore, Temecula, CA, United States) and rabbit polyclonal anti-vesicular GABA transporter (VGAT) antibody (1:500, Gene Tex, Los Angeles, CA, United States). After washing three times with PBS, the brain sections were incubated with an appropriate species-specific secondary antibody for 1 h−Alexa Fluor 488-conjugated goat anti-mouse immunoglobin (Ig) G (1:200, Abcam) or Alexa Fluor 647-conjugated goat anti-rabbit IgG (1:200, Merck-Millipore)−and then with 4′,6-diamidino-2-phenylindole dihydrochloride (DAPI) solution (1:500, Dojindo, Kamimashiki, Japan). After three washes with PBS, the sections were mounted using Fluoromount (Diagnostic BioSystems, Pleasanton, CA, United States).

### Quantification and Imaging

#### Tissue Observation

We observed samples under a confocal microscope (Zeiss LSM 800, Carl Zeiss, Jena, Germany) with a 20 × (NA 0.80) or 63 × (NA 1.4) objective lens (LSM 800, Carl Zeiss). Images were captured using a charge-coupled device camera (Axio-Cam MRm, Carl Zeiss), transferred to a computer, and analyzed using ZEN software (Carl Zeiss).

#### Definition of Layers of the Cerebral Cortex and Hippocampus

**FIGURE 1:**
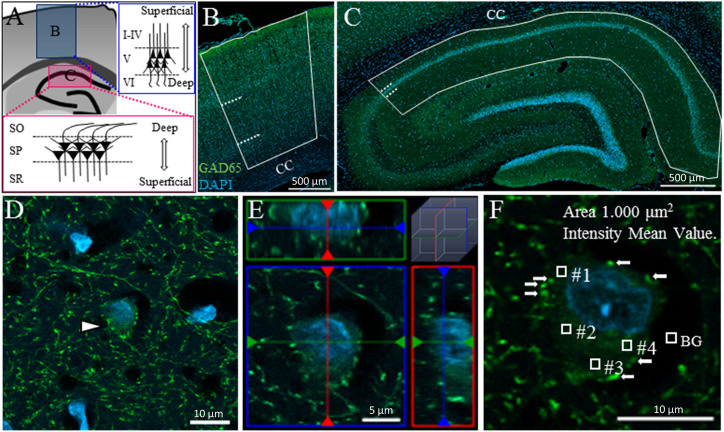
Measuring GAD65 staining intensity in somata in the cerebral cortex and hippocampus. Schematic drawing of a coronal section of an adult rat brain showing the cerebral cortex (**A:** upper inset **B**) and hippocampus (**A:** lower inset **C**). The cerebral cortex and hippocampus were each divided into three layers. Solid lines and dashed lines indicate the region of interest and the boundaries between layers, respectively, in the cerebral cortex **(B)** and hippocampus **(C)**. The area of GAD65 expression in the soma was surrounded by synaptic buttons in the 2D (**D:** arrowhead) and 3D images (**E:** ortho-image from the *x*, *y*, and *z* planes). The level of GAD65 expression was calculated by subtracting the background (BG) intensity from the average intensity of four squares **(F)**. CC, corpus callosum.

In the cerebral cortex, we examined the primary motor area, primary somatosensory area and posterior parietal association area. Since we could not reliably divide these cortical areas, we did not analyze the differences among these cerebral cortical areas. For the same reason, we also divided the cortex into three layers (Layer II, III, and IV are not distinguished) the superficial (II–IV), pyramidal (V), and deep (VI) layers (Figure 1A: upper inset; Figure 1B). Layer I was not included in the superficial layer in this study, as this layer contains very few cells of GABAergic subtypes other than CR^+^ and nNOS^+^ neurons (Kubota et al., 2011).

In the hippocampus, we examined the CA1 region in the dorsal hippocampus. The hippocampal CA1 region was divided into three layers: the superficial [stratum radiatum (SR)], pyramidal [stratum pyramidale (SP)], and deep [stratum oriens (SO)] layers (Figure 1A: lower inset; Figure 1C).

#### Evaluation of GAD65 Expression in the Soma Using Serial *z*-Stack Sections

We measured the intensity of GAD65 expression in somata but not in synaptic buttons. In two-dimensional (2D) images, distinguishing GAD65 expression in the soma from that in synaptic buttons is technically difficult. Figure 1D shows an example of a GAD65-positive (GAD65^+^: green) cell in the hippocampus (arrowhead). The nucleolus was stained with DAPI (blue). GAD65 expression was observed in the soma and perisomatic region. Such perisomatic staining shows synaptic buttons (axon terminals) as somatic inputs because GAD65 is localized to axon terminals but not to dendrites (Kanaani et al., 2002).

To distinguish the GAD65 staining in somata from that in axon terminals, we followed the criteria proposed by Jinno and Kosaka (2009). According to their criteria, we captured serial stacks of optical sections at 1-μm intervals using confocal microscopy with a *z*-stack system, as cell bodies (>2 μm) appear in multiple adjacent sections, whereas buttons (<2 μm) do not appear in more than two optical sections. To validate these criteria for GAD65 staining, we reconstructed the GAD65^+^ cell in a three-dimensional (3D) image (Figure 1E) and then confirmed that these criteria can be used to successfully distinguish between GAD65 expression in the soma and that in synaptic buttons. The reconstruction of 3D images has been described previously (Kajita et al., 2017).

To measure GAD65 intensity in the soma, we randomly set four squares within each soma, avoiding the nucleus and buttons (arrows), and measured the average intensity within those squares (0, no signal; 255, maximum level; Figure 1F). The average intensity over the four squares was used as the datapoint for GAD65 intensity after the subtraction of background signals. To avoid measuring out-of-focus cells, we selected only cells with clear DAPI staining. To measure GAD65 intensity in the synaptic buttons, we selected the 50 synaptic buttons from VGAT staining, and the average intensity was used as the data point after the subtraction of background signals. The density of VGAT was measured in unit area (/100 μm^2^). The average density of 25 unit areas was used as the datapoint for VGAT density per brain. Data were collected in Excel (Microsoft, Redmond, WA, United States) for further analysis. All data were calculated with the truncation of numbers beyond the third or fourth decimal place.

### Statistical Analysis

We used IBM SPSS Statistics for Windows software (version 21.0 [released 2012], IBM Corp., Armonk, NY, United States) for statistical analysis and performed Welch’s *t*-test, Mann–Whitney *U* Test, two-way analysis of variance (ANOVA) followed by Bonferroni’s *post hoc* tests, and Pearson’s correlation test. Values are presented as the mean ± standard error of the mean (SEM). Groups were compared using thresholds for significance of 0.05 and 0.01.

## Results

### GAD65 Expression Levels Among GABAergic Subtypes and Layers of the Cerebral Cortex

**FIGURE 2:**
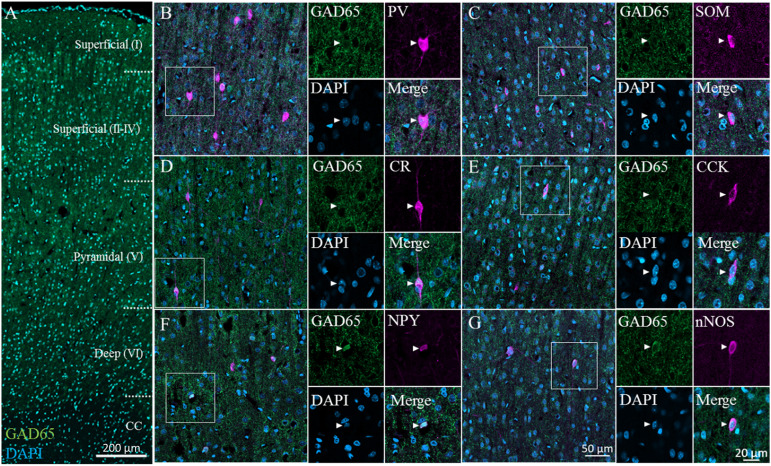
GAD65 expression in each GABAergic subtype in the cerebral cortex. Double immunofluorescence image of GAD65 (green) and DAPI (blue) signals **(A)**. Triple immunofluorescence images showing GAD65 expression (green); positive signals for PV, SOM, CR, CCK, NPY, or nNOS (magenta); and DAPI staining (blue) in the superficial (II–IV) layer **(B–G)**. White squares indicate the regions enlarged in the panels on the right, and arrowheads indicate somata.

We immunostained for GAD65 in the cerebral cortex; a typical image captured via confocal microscopy is shown in Figure 2A (GAD65: green; DAPI: blue). GAD65 expression was detected throughout the imaged region, except in the corpus callosum. All six GABAergic subtypes were detected across layers II–VI. On the other hand, only CR^+^ and nNOS^+^ neurons were detected in layer I. We present representative high-magnification images of the six GABAergic subtypes in the superficial (II–IV) layer in Figures 2B–G, which show triple signals of GAD65 immunofluorescence (green), the GABAergic subtype (magenta), and DAPI staining (blue).

**FIGURE 3:**
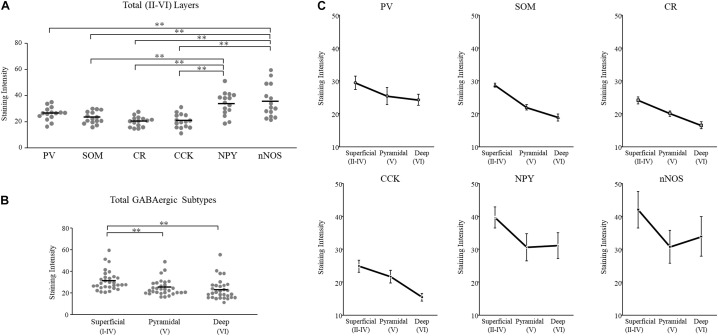
GAD65 expression in each GABAergic subtype across layers of the cerebral cortex (*n* = 5 brains). The average intensity of GAD65 immimostaining among six GABAergic subtypes **(A)**. The average intensity of GAD65 immunostaining among the superficial (II–IV), pyramidal (V), and deep (VI) layers **(B)**. The average intensity of GAD65 immunostaining across layers of the cerebral cortex among six GABAergic subtypes **(C)**. Error bars indicate SEM. Two-way ANOVA followed by Bonferroni’s tests was used to compare the expression level. ^∗∗^*p* < 0.01.

We plotted GAD65 intensity among the six subtypes present in those three layers in Figure 3. Further details are provided in Table 1.

**TABLE 1:** GAD65 Expression in Cerebral Cortical Layers Based on GAD65 Staining Intensity.

**Layer**	**Subtypes**	Mean Intensity of GAD65*	Numbers of Cells in each GAD65-Intensity Range**									**Subtypes ratio (%)**
			**0–10**	**11–20**	**21–30**	**31–40**	**41–50**	**51–60**	**61–70**	**71–255**	**Total**	
Superficial (I)	CR	27.95 ± 3.15	2	9	11	4	2	0	1	0	29	52.72
	nNOS	70.04 ± 11.32	0	1	3	5	3	6	0	8	26	47.27
Superficial (II–IV)	PV	29.34 ± 2.09	0	47	97	58	41	10	2	2	257	25.90
	SOM	28.63 ± 0.60	10	51	64	38	19	7	2	1	192	19.35
	CR	24.00 ± 1.09	12	76	67	32	15	3	0	0	205	20.66
	CCK	24.77 ± 1.89	6	37	32	26	1	1	0	0	103	10.38
	NPY	39.63 ± 3.27	2	13	41	37	21	14	12	3	143	14.41
	nNOS	41.94 ± 5.61	1	15	23	26	10	7	7	3	92	9.27
Pyramial (V)	PV	25.40 ± 2.59	4	70	118	45	10	0	0	0	247	38.35
	SOM	21.90 ± 0.82	12	68	55	15	2	0	0	0	152	23.60
	CR	20.02 ± 0.82	8	36	22	5	1	0	0	0	72	11.18
	CCK	21.57 ± 1.96	5	20	16	5	2	0	0	0	48	7.45
	NPY	30.54 ± 4.08	2	23	34	11	9	3	2	1	85	13.19
	nNOS	30.70 ± 4.96	0	12	13	4	6	4	0	1	40	6.21
Deep (VI)	PV	24.19 ± 1.68	6	52	86	42	4	0	0	0	190	31.09
	SOM	18.87 ± 1.09	25	73	41	9	1	0	0	0	149	24.38
	CR	16.46 ± 1.10	19	44	13	2	0	1	0	0	79	12.92
	CCK	15.40 ± 1.20	14	21	16	1	1	0	0	0	53	8.67
	NPY	31.05 ± 3.91	3	23	31	25	10	4	3	4	103	16.85
	nNOS	33.83 ± 6.01	1	5	13	6	6	3	3	0	37	6.05

*Mean of five brains (Five rats).

**Total cells from five brains (Total 2302 cells).

#### GAD65 Expression Was High in NPY^+^ and nNOS^+^ Neurons and Low in PV^+^, SOM^+^, CR^+^, and CCK^+^ Neurons Across Layers of the Cerebral Cortex

To examine subtype- and layer-related specificity, we statistically analyzed the GAD65 staining intensity using two-way ANOVA (subtype × layer). The statistical analysis showed significant main effects due to the subtype and layer type (subtypes: *p* = 0.000; layers: *p* = 0.000), but their interaction was not significant.

To compare the GAD65 staining intensity among the six subtypes, we performed multiple comparison analysis using Bonferroni’s *post hoc* method. The GAD65 staining intensity was significantly higher in NPY^+^ and nNOS^+^ neurons than in other subtypes (NPY^+^ vs. SOM^+^, *p* = 0.001; vs. CR^+^, *p* = 0.000; vs. CCK^+^, *p* = 0.000), (nNOS^+^ vs. PV^+^, *p* = 0.006; vs. SOM^+^, *p* = 0.000; vs. CR^+^, *p* = 0.000; vs. CCK^+^, *p* = 0.000; Figure 3A). The difference between NPY^+^ and PV^+^ neurons was not quite significant. In the superficial (I) layer, Welch’s *t*-test revealed that the GAD65 staining intensity was significantly higher in nNOS^+^ neurons than in CR^+^ neurons (*p* = 0.018).

Based on these statistical analysis, we classified GABAergic subtypes into two groups: high GAD65 (NPY^+^ and nNOS^+^) and low GAD65 (PV^+^, SOM^+^, CR^+^, and CCK^+^) neurons in the cerebral cortex.

#### GAD65 Expression Was Higher in the Superficial Layer Than in Other Layers of the Cerebral Cortex

To compare the GAD65 staining intensity among layers of the cerebral cortex, we performed multiple comparison analysis using Bonferroni’s *post hoc* method. The GAD65 staining intensity was significantly higher in the superficial (II–IV) layer than in the pyramidal (V; *p* = 0.002) and deep (VI; *p* = 0.000) layers (Figure 3B).

Based on these results, GAD65 expression is higher in the superficial layer than in other layers of the cerebral cortex. The GAD65 staining intensity among layers in each subtype was shown in Figure 3C. In the cerebral cortex there was no significant interaction (subtype × layer). We did not perform statistical analysis among the six subtypes in each three layer, or among three layers in each six subtype.

### GAD65 Expression Among GABAergic Subtypes Across Layers of the Hippocampus

**FIGURE 4:**
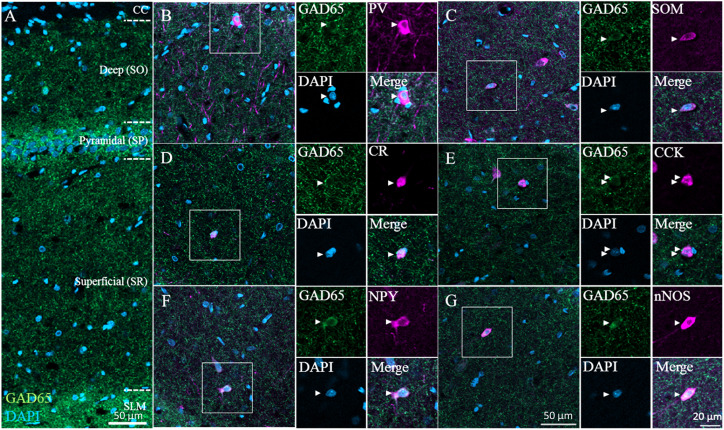
GAD65 expression in each GABAergic subtype in the hippocampus. Double immunofluorescence image showing GAD65 (green) and DAPI (blue) signals **(A)**. Triple immunofluorescence images showing GAD65 expression (green); positive signals for PV, SOM, CR, CCK, NPY, or nNOS (magenta); and DAPI staining (blue) in the superficial (SR) layer **(B–G)**. White squares indicate the regions enlarged in the panels on the right, and arrowheads indicate somata. CC, corpus callosum; SO, stratum oriens; SP, stratum pyramidale; SR, stratum radiatum; and SLM, stratum lacunosum-moleculare.

Next, we immunostained for GAD65 in the CA1 region of the hippocampus; a typical image from confocal microscopy can be seen in Figure 4A (GAD65: green; DAPI: blue). GAD65 expression was detected across the entire region excluding the corpus callosum. We detected all six GABAergic subtypes, and high-magnification examples of each subtype as observed in the superficial (SR) layer can be found in Figures 4B–G, which show triple signals of GAD65 immunofluorescence (green), the GABAergic subtype (magenta), and DAPI staining (blue).

**FIGURE 5:**
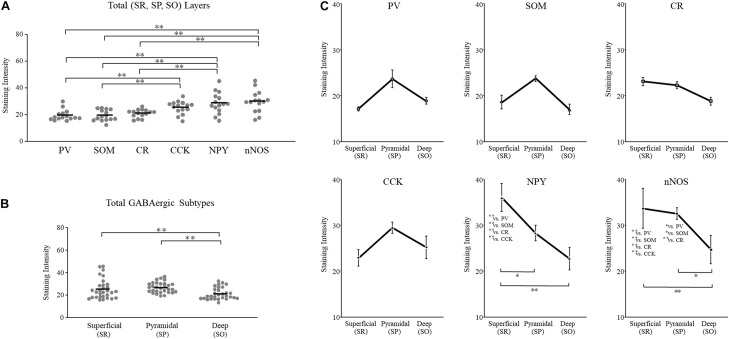
GAD65 expression in each GABAergic subtype across layers of the hippocampus (*n* = 5 brains). The average intensity of GAD65 immunostaining among six GABAergic subtypes **(A)**. The average intensity of GAD65 immunostaining among the superficial (SR), pyramidal (SP), and deep (SO) layers **(B)**. The average intensity of GAD65 immunostaining across layers of the cerebral cortex among six GABAergic subtypes **(C)**. SO, stratum oriens; SP, stratum pyramidale; and SR, stratum radiatum. Error bars indicate SEM. Two-way ANOVA followed by Bonferroni’s tests was used to compare the expression level. ^∗^*p* < 0.05. ^∗∗^*p* < 0.01.

We plotted the GAD65 staining intensity among the six subtypes and three layers in Figure 5. Further details are provided in Table 2.

**TABLE 2:** GAD65 Expression in Hippocampal Layers Based on GAD65 Staining Intensity.

**Layer**	**Subtypes**	Mean Intensity of GAD65*	Numbers of Cells in each GAD65-Intensity Range**									**Subtypes ratio (%)**
			**0–10**	**11–20**	**21–30**	**31–40**	**41–50**	**51–60**	**61–70**	**71–255**	**Total**	
Superficial (SR)	PV	17.11 ± 0.40	26	30	19	4	2	0	0	0	81	7.97
	SOM	18.59 ± 1.50	12	34	14	6	1	1	0	0	68	6.69
	CR	23.08 ± 0.85	44	131	91	44	21	5	1	1	338	33.26
	CCK	22.87 ± 1.82	19	115	65	31	13	8	0	0	251	24.70
	NPY	36.03 ± 3.06	5	17	39	44	26	14	9	5	159	15.64
	nNOS	33.66 ± 4.36	0	30	34	22	14	9	5	5	119	11.71
Pyramidal (SP)	PV	23.65 ± 1.93	31	158	127	76	17	2	1	2	414	34.10
	SOM	23.75 ± 0.66	3	21	22	7	4	0	0	0	57	4.69
	CR	22.25 ± 0.76	28	128	100	37	11	1	0	1	306	25.20
	CCK	29.44 ± 1.21	2	24	45	34	15	4	0	0	124	10.21
	NPY	28.25 ± 1.70	13	55	58	35	18	12	2	0	193	15.89
	nNOS	32.53 ± 1.29	1	8	53	33	17	6	1	1	120	9.88
Deep (SO)	PV	18.77 ± 0.86	58	153	84	24	5	0	0	0	324	21.92
	SOM	16.96 ± 1.13	106	303	106	22	3	1	0	0	541	36.60
	CR	18.80 ± 1.09	34	126	53	17	2	0	0	0	232	15.69
	CCK	25.18 ± 2.42	8	35	51	27	8	1	1	0	131	8.86
	NPY	22.69 ± 2.42	23	83	60	18	7	4	0	2	197	13.32
	nNOS	24.70 ± 3.08	1	23	16	9	2	2	0	0	53	3.58

*Mean of five brains (Five rats).

**Total cells from five brains (Total 3708 cells).

#### GAD65 Expression Was Layer Specific and High in NPY^+^, nNOS^+^, and CCK^+^ Neurons but Low in PV^+^, SOM^+^, and CR^+^ Neurons

To examine subtype- and layer-related specificity, we statistically analyzed the GAD65 staining intensity using two-way ANOVA (subtype × layer). The statistical analysis indicated significant main effects due to the subtype and layer type (subtypes: *p* = 0.000; layers: *p* = 0.000). The interaction was also significant (subtype × layer: *p* = 0.002).

To compare the GAD65 staining intensity among the six subtypes, we performed multiple comparison analysis using Bonferroni’s *post hoc* method. The GAD65 staining intensity was significantly higher in NPY^+^, nNOS^+^, and CCK^+^ neurons than in other subtypes (NPY^+^ vs. PV^+^, *p* = 0.000; vs. SOM^+^, *p* = 0.000; vs. CR^+^, *p* = 0.000; nNOS^+^ vs. PV^+^, *p* = 0.000; vs. SOM^+^, *p* = 0.000; vs. CR^+^, *p* = 0.000; CCK^+^ vs. PV^+^, *p* = 0.006; vs. SOM^+^, *p* = 0.005; Figure 5A). No significant difference was found between CCK^+^ and CR^+^ neurons.

However, these main effects were qualified by a significant interaction between subtype and layer type. Therefore, we compared the GAD65 staining intensity among the six subtypes for each layer using Bonferroni’s *post hoc* tests. In the superficial (SR) layer, the GAD65 staining intensity was significantly higher in NPY^+^ (NPY^+^ vs. PV^+^, *p* = 0.000; vs. SOM^+^, *p* = 0.000; vs. CR^+^, *p* = 0.000; vs. CCK^+^, *p* = 0.000) and nNOS^+^ neurons (nNOS^+^ vs. PV^+^, *p* = 0.000; vs. SOM^+^, *p* = 0.000; vs. CR^+^, *p* = 0.005; vs. CCK^+^, *p* = 0.004; Figure 5C). In the pyramidal (SP) layer, the GAD65 staining intensity was significantly higher in nNOS^+^ neurons than in all other subtypes (nNOS^+^ vs. PV^+^, *p* = 0.033; vs. SOM^+^, *p* = 0.037; vs. CR^+^, *p* = 0.007). By contrast, in the deep (SO) layer, no significant differences were found.

Based on these statistical analysis, we classified GABAergic subtypes into two groups: high GAD65 (NPY^+^, nNOS^+^, and CCK^+^) and low GAD65 (PV^+^, SOM^+^, and CR^+^) neurons in the hippocampus. In contrast to the cerebral cortex, these high expression levels are indicative of layer specificity. GAD65 expression in the superficial layer (SR) was higher in NPY^+^ and nNOS^+^ neurons, and GAD65 expression in the pyramidal layer (SP) was higher in nNOS^+^ neurons than in other subtypes. In the deep layer (SO), GAD65 expression tended to be high in CCK^+^ neurons, but no significant differences were noted.

#### GAD65 Expression Was Subtype Specific and Higher in the Superficial and Pyramidal Layers Than in the Deep Layer of the Hippocampus

To compare the GAD65 staining intensity among hippocampal layers, we performed multiple comparison analysis using Bonferroni’s *post hoc* method. The GAD65 staining intensity was significantly higher in the superficial (SR) and pyramidal (SP) layers than in the deep (SO) layer (SR vs. SO, *p* = 0.002; SP vs. SO, *p* = 0.000; Figure 5B).

As described above, these main effects were qualified by a significant interaction between the subtype and layer type. Therefore, we compared the GAD65 staining intensity among layers for each subtype using Bonferroni’s *post hoc* tests. In NPY^+^ neurons, the GAD65 staining intensity was significantly higher in the superficial (SR) layer than in the other (SP and SO) layers (SR vs. SP, *p* = 0.021; SR vs. SO, *p* = 0.000; Figure 5C). In nNOS^+^ neurons, the GAD65 staining intensity was significantly higher in the superficial (SR) and pyramidal (SP) layers than in the deep (SO) layer (SR vs. SO, *p* = 0.006: SP vs. SO, *p* = 0.020). In PV^+^, SOM^+^, CR^+^, and CCK^+^ neurons, the GAD65 staining intensity was not significantly different across layers.

Based on these results, GAD65 expression was higher in the superficial (SR) and pyramidal (SP) layers than in the deep (SO) layer. However, this laminar distribution was subject to subtype specificity. In NPY^+^ neurons, GAD65 expression was high in the superficial (SR) layer. In nNOS^+^ neurons, GAD65 expression was high in the superficial (SR) and pyramidal (SP) layers. In PV^+^, SOM^+^, and CCK^+^ neurons, no significant differences were observed, but GAD65 expression tended to be high in the pyramidal (SP) layer.

### GAD65 Expression in Different Subtypes Was Positively Correlated Between Colchicine-Treated and Control Brains

**FIGURE 6:**
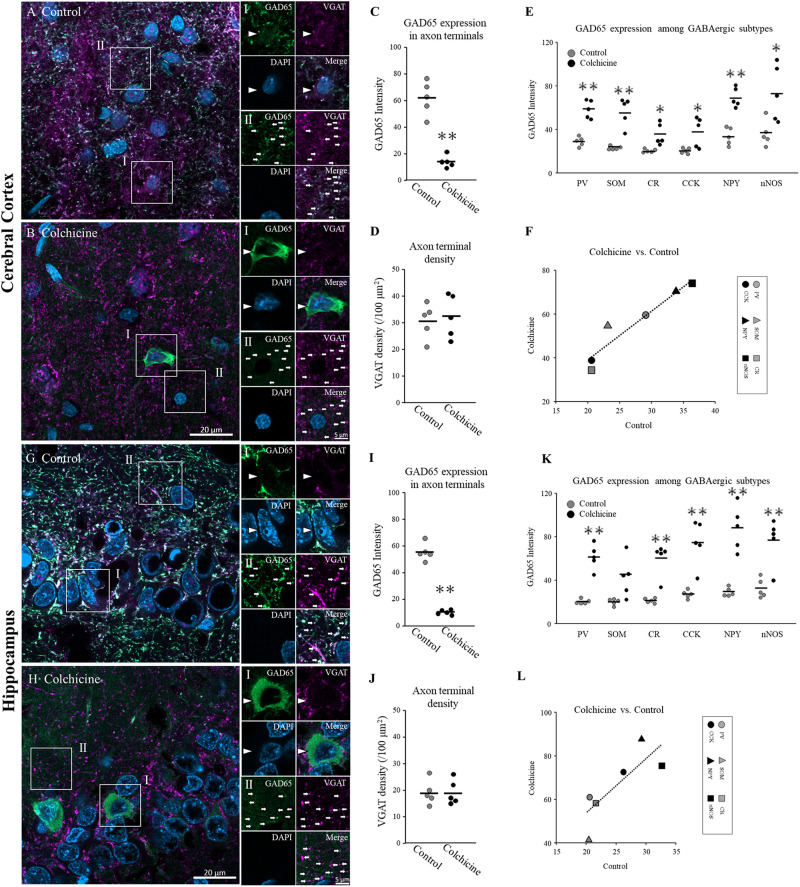
GAD65 expression in the cerebral cortex and hippocampus of colchicine-treated rats (*n* = 5 brains). Triple immunofluorescence image showing GAD65 (green), VGAT (magenta), and DAPI (blue) signals (cerebral cortex: **A,B**; hippocampus: **G,H**). White squares (I: soma; II: neuropil) indicate the regions enlarged in the panels on the right, and arrowheads and arrows indicate somata and axon terminals, respectively. The average GAD65 immunostaining intensity in axon terminals (neuropil) in cerebral cortical layers **(C)** and hippocampal layers **(I)**. The density of VGAT immunostaining per unit area in cerebral cortical layers **(D)** and hippocampal layers **(J)**. The average GAD65 immunostaining intensity in soma in cerebral cortical layers **(E)** and hippocampal layers **(K)** among six GABAergic subtypes. Correlations in GAD65 staining intensities in six GABAergic subtypes in the cerebral cortex **(F)** and hippocampus **(L)** between the colchicine-injected group and the control group. Black bars indicate the mean value of five brains **(C–E,I–K)**. Error bars indicate SEM. Welch’s *t*-test revealed was used for **(C,E,D,I,K,J)**. Mann–Whitney *U* Test was used for **(K)**. Pearson’s correlation analysis was used for **(F,L)**. ^∗^*p* < 0.05. ^∗∗^*p* < 0.01.

We examined GAD65 expression in somata but not in axon terminals. Investigating GAD65 expression in axon terminals is difficult because the SOM, NPY, and nNOS proteins cannot be clearly detected in axon terminals under confocal microscopy. To detect GAD65 expression throughout the cytoplasm including in axon terminals, we injected colchicine, an inhibitor of microtubule polymerization, into the lateral ventricle to block axonal transport. At 2 days after colchicine injection, the GAD65 staining (green) intensity increased in somata in the cerebral cortex (Figures 6A-I, B-I, arrowhead) and hippocampus (Figures 6G-I, H-I, arrowhead). On the other hand, the intensity of GAD65 decreased in the neuropil region of the cerebral cortex (Figures 6A-II, B-II, arrows) and hippocampus (Figures 6G-II, H-II, arrows). Welch’s *t*-test showed the intensities of GAD65 clusters were significantly lower in colchicine-injected brains (Figure 6C, cerebral cortex: *p* = 0.001; Figure 6I, hippocampus: *p* = 0.000). In additions, the number of VGAT clusters (magenta) were not changed in colchicine-injected brains (Figure 6D, cerebral cortex; Figure 6J, hippocampus). VGAT is localized in inhibitory axon terminal (Chaudhry et al., 1998), suggesting that GAD65 was not transported to axon terminals and instead accumulated in somata. In these brains, we measured GAD65 expression in the six subtypes in the cerebral cortex (Figure 6E) and hippocampus (Figure 6K) and compared the results with control brains. In colchicine-injected brains, the intensities of GAD65 staining in somata were dramatically increased, but their distributions throughout the somata did not appear to show the major changes. Therefore, we determined that same quantitative method as control is applicable in them. GAD65 expression was compared using the mean value of the three layers in both colchicine-injected and control brains. Welch’s *t*-test showed the intensities of GAD65 in somata were significantly higher in colchicine-injected brains (Figure 6E, cerebral cortex: PV, *p* = 0.000; SOM, *p* = 0.004; CR, *p* = 0.026; CCK, *p* = 0.049; NPY, *p* = 0.000; and nNOS, *p* = 0.029; Figure 6K, hippocampus: PV, *p* = 0.001; CCK, *p* = 0.005; NPY, *p* = 0.003; and nNOS, *p* = 0.006). The data of CR did not show normal distribution, and was detected the significant difference using Mann–Whitney *U* Test (*p* = 0.008). There was no significant difference in SOM^+^ cells between colchicine-injected and control brains. Further details are provided in Table 3. Pearson’s test showed significant positive correlations in the expression profile between colchicine-injected and control brains (Figure 6F, cerebral cortex: *r* = 0.780, *p* = 0.003; Figure 6L, hippocampus: *r* = 0.889, *p* = 0.000).

**TABLE 3:** GAD65 Expression in (A) Cerebral Cortical (B) Hippocampal layers based on GAD65 staining intensity in colchicine-injected rats.

(A) Cerebral Cortex														
	**Subtypes**	Mean Intensity of GAD65*	Numbers of Cells in each GAD65-Intensity Range**											
			**0–10**	**11–20**	**21–30**	**31–40**	**41–50**	**51–60**	**61–70**	**71–80**	**81–90**	**91–100**	**101–255**	**Total**

Colchicine	PV	60.26 ± 3.71	0	1	7	14	24	23	21	15	6	3	0	114
	SOM	55.93 ± 5.82	2	7	12	3	3	5	4	4	8	10	10	68
	CR	35.65 ± 4.41	3	3	9	18	9	13	13	8	9	9	3	68
	CCK	37.51 ± 6.11	0	1	4	15	15	10	8	7	0	0	0	60
	NPY	69.49 ± 3.98	0	0	0	10	25	15	30	5	5	10	25	125
	nNOS	73.62 ± 11.61	0	0	8	0	6	12	3	3	5	10	18	65
Control	PV	29.07 ± 1.91	17	50	67	39	7	7	1	0	0	0	0	188
	SOM	23.10 ± 0.70	21	66	22	5	1	0	0	0	0	0	0	115
	CR	20.59 ± 0.69	9	27	18	6	3	0	0	0	0	0	0	63
	CCK	20.58 ± 1.13	7	17	14	9	0	0	0	0	0	0	0	47
	NPY	33.85 ± 3.55	0	18	23	20	5	4	5	1	0	0	0	76
	nNOS	36.38 ± 5.25	0	5	14	11	10	8	4	1	1	0	0	54

**(B) Hippocampus**														

	**Subtypes**	Mean Intensity of GAD65*	Numbers of Cells in each GAD65-Intensity Range***											
			**0–10**	**11–20**	**21–30**	**31–40**	**41–50**	**51–60**	**61–70**	**71–80**	**81–90**	**91–100**	**101–255**	**Total**

Colchicine	PV	61.57 ± 5.12	0	15	17	25	21	12	14	10	6	6	28	154
	SOM	42.80 ± 8.16	18	11	23	17	6	10	3	3	2	1	6	100
	CR	59.98 ± 6.62	5	17	16	17	9	9	4	5	4	3	20	109
	CCK	73.79 ± 9.21	3	4	5	6	6	8	5	9	3	3	34	86
	NPY	88.11 ± 9.23	0	3	5	0	5	3	4	7	2	6	37	72
	nNOS	75.51 ± 9.51	5	8	11	12	6	8	11	3	7	6	32	109
Control	PV	20.41 ± 0.97	17	63	43	16	3	0	0	0	0	0	0	142
	SOM	20.19 ± 1.26	10	41	37	9	5	1	0	0	0	0	0	103
	CR	21.49 ± 0.95	16	52	34	16	5	1	1	0	0	0	0	125
	CCK	27.09 ± 1.60	5	26	31	21	5	5	1	0	0	0	0	94
	NPY	29.27 ± 1.81	6	23	25	16	10	4	2	1	1	0	0	88
	nNOS	32.64 ± 3.97	1	17	32	20	11	7	3	1	1	1	1	95

*Mean of five brains (Five rats).

**Total cells from five brains (Colchicine: 529 cells, Control: 543 cells).

***Total cells from five brains (Colchicine: 630 cells, Control: 647 cells).

Based on these results, GAD65 expression in the soma and cytoplasm exhibited similar tendencies across all GABAergic subtypes, and the level of GAD65 expression in the soma can be used as a proxy for the level of GAD65 expression in the cytoplasm for all GABAergic subtypes.

## Discussion

In this study, we compared GAD65 expression among six GABAergic subtypes and found that GABAergic subtypes fell into two classes for each brain region from statistical analysis: a high-expression group (NPY^+^ and nNOS^+^) and a low-expression group (PV^+^, SOM^+^, CR^+^, and CCK^+^) in the cerebral cortex, and a high-expression group (CCK^+^, NPY^+^, and nNOS^+^), and a low-expression group (PV^+^, SOM^+^, and CR^+^) in the hippocampus. There was a difference in the laminar distribution of GAD65 expression between the cortical and hippocampal layers.

Additionally, GAD65 expression across all GABAergic subtypes were significantly and positively correlated between control and colchicine-treated rats.

### nNOS^+^ and NPY^+^ Neurons Express GAD65 at Higher Levels Than Do the Other Subtypes

Our results revealed that nNOS^+^ and NPY^+^ neurons express GAD65 at particularly high levels, whereas some major subtypes (PV^+^/SOM^+^/CR^+^) express GAD65 at low levels in the cerebral cortex and hippocampus. This result is consistent with previous reports of PV^+^ neurons having lower GAD65 expression than non-PV neurons in the mouse hippocampus (Fukuda et al., 1997). Candidates of these non-PV neurons having high GAD65 may include the nNOS^+^ and NPY^+^ neurons based on our findings. There was another previous report comparing synaptic-GAD65 and GAD67 expression among PV^+^ basket neurons, PV^+^ chandelier neurons and Calbindin-positive (CB^+^) basket neurons in the monkey prefrontal cortex (Fish et al., 2011). They shows the higher synaptic expression of GAD65 in PV^+^ basket neurons than in PV^+^ chandelier neurons, and the same level of synaptic expression of GAD65 in PV^+^ basket neurons as in CB^+^ basket neurons. Because of different classification of GABAergic subtypes, it would be necessary to clarify distinct patterns of GAD65 expressions in synapse levels at multiple GABAergic subtype.

Neuropeptide Y is a neuromodulator with anti-seizure activity (Erickson et al., 1996; Baraban et al., 1997; Vezzani et al., 1999). Its anti-epileptic functions occur during the pre- and post-synaptic inhibition of excitatory neurons via Y1, Y2, and Y5 receptors (Baraban, 2004). NPY belongs to the G-protein-coupled inwardly rectifying potassium receptor family (Paredes et al., 2003). The functions of neuromodulator/GABA co-release remain unclear, but generally, GABA transmission contributes to fine-tuning of the synaptic effects of neuromodulators (Horvath et al., 2001; Yu et al., 2015; Tritsch et al., 2016). Many NPY^+^ neurons are excited by GABA in the hilus of the hippocampus (Fu and van den Pol, 2007), suggesting that NPY^+^ cells are activated by GABA, which results in the increased release of not only GABA but also the co-expressed inhibitory neuromodulator NPY. Based on these reports, we suggest that the neural inhibitory functions of NPY are regulated by GAD65-dependent GABA release in pathological situations.

The pharmacological functions of nitric oxide (NO) in epileptic seizures remain controversial, but NO has been considered to act as a mediator of neurotoxic effects in several reports (Dawson et al., 1991; Marangoz et al., 1994; Penix et al., 1994; Schuman and Madison, 1994). nNOS has the function of evoking the release of several neurotransmitters, including acetylcholine, catecholamines, neuroactive amino acids and GABA (Kahn et al., 1997a,b). NO-evoked neurotransmitter release is mediated by two distinct release systems, a Ca^2+^-dependent system and the reverse process of a Na^+^-dependent carrier-mediated transport system (Garthwaite, 1991; Kuriyama and Ohkuma, 1995). NO has effects of decreasing GABA transaminase activity and increasing GABA expression (Jayakumar et al., 1999; Vega Rasgado et al., 2018). The relationship between presynaptic NO release and GABA remains largely unknown, but as with NPY, NO, and GABA release may be related to the regulation of overexcited neurons in some pathological states. The high concentrations of GAD65 in nNOS^+^ and NPY^+^ neurons may be needed to support a rapid response to neuromodulators when necessary.

Additionally, SOM is an anti-epileptic neuropeptide in the hippocampus (Boehm and Betz, 1997; Schweitzer et al., 1998; Tallent and Siggins, 1999). GABA-mediated activation of NPY^+^ neurons may increase the release of SOM (Fu and van den Pol, 2007), as SOM/NPY co-expression has been observed in a subset of GABAergic neurons in the hippocampus (Köhler et al., 1987). However, GAD65 expression was not high in SOM^+^ neurons according to our results, indicating that the release of SOM is not associated with GAD65-dependent GABA release.

### Laminar Distributions of GAD65 in the Cerebral Cortex and Hippocampus

In the cerebral cortex, we found that GAD65 expression is higher in the superficial layer than in the other layers for all GABAergic subtypes. Pyramidal neurons project their dendrites into the superficial layer to receive amino-acid or monoamine signals from other parts of the cortex or extra-cortical regions. Inhibitory dendritic signals from local GABAergic neurons are essential for mediating these inputs and maintaining a normal neural state (Northoff and Mushiake, 2020). Therefore, high GAD65 expression in neurons in the superficial layer may play a crucial role in suppressing cortical over-excitation. This idea is consistent with a report stating that local GABAergic inhibition drives the elasticity of ictal progression and that cortical seizures occur in superficial layers prior to deep layers during lateral seizure spread (Wenzel et al., 2017). Additionally, GAD65 expression was found to be higher in the pyramidal layer of the visual cortex of 1-week-old cats (Mower and Guo, 2001), but higher expression was detected in the superficial layer in adults (Guo et al., 1997). This pattern change may reflect flexible regulation of GAD65 for maintaining a normal state during the growth of neural circuits.

In the hippocampus, we observed that GAD65 expression was higher in the superficial layer than in the deep layer only in nNOS^+^ and NPY^+^ neurons. Dendrites of hippocampal pyramidal neurons in the superficial layer receive glutamatergic Schaffer collateral inputs from the CA3 region. In the hippocampal CA1 region, GABAergic neurons expressing nNOS/NPY are known as Ivy cells (Price et al., 2005; Tricoire and Vitalis, 2012). Ivy cells provide widespread synaptic and extra-synaptic slow inhibition of the dendrites of CA1 pyramidal neurons (Fuentealba et al., 2008; Lapray et al., 2012). The number of Ivy cells was significantly reduced in the hippocampus of pilocarpine-induced epileptic rats. Interestingly, the number of PV^+^ neurons was not changed in this model (Orbán-Kis et al., 2015). These findings suggest that nNOS^+^ and NPY^+^ neurons in the superficial layer may play a critical role in suppressing ictal progression in the hippocampus. Same as nNOS^+^ and NPY^+^ neurons, in PV^+^, SOM^+^, CR^+^ and CCK^+^ neurons, they also showed the tendency of distinct GAD65 expression among layers. These results are consistent with a previous report stating that both PV^+^ and non-PV neurons exhibit distinct laminar distribution of GAD65 in the deep and pyramidal layer (Fukuda et al., 1997).

### Measurement of GAD65 Expression in the Soma

We noted that strong GAD65 fluorescence often accumulated in the perinuclear region. This localization may reflect GAD65 protein undergoing a hydrophobic posttranslational modification and becoming anchored to the cytosolic face of the Golgi membranes (Kanaani et al., 2008).

Based on this feature, we considered it inappropriate to compare GAD65 expression through measurement of its mean intensity inside a traced outline of the soma, as described in previous report (Jinno and Kosaka, 2009). GABAergic neurons have different soma size depending on subtypes (González-Albo et al., 2001). This affects the ratio of the Golgi membrane occupying the soma differs among GABAergic subtypes. To avoid this problem, we quantified GAD65 expression from the average intensity of four squares (details are provided in the section “Materials and Methods”); this method is considered less susceptible to the effects of soma size.

### GAD65 Expression in the Soma Can Be Used as a Proxy for Its Level in the Cytoplasm

GAD65 localizes to both the soma and axon terminal, and thus, the amount of GAD65 in the soma does not necessarily reflect the total number of GAD65 molecules throughout the cytoplasm. As reported previously, there are two possible drivers of high GAD65 expression in the soma: a high synthesis rate or slow transport to the axon terminals (Esclapez et al., 1993). The inhibition of axonal transport by colchicine enhances GAD65 immunoreactivity in the soma (Wang et al., 2014). We found that GAD65 expression in the soma across neuronal subtypes was significantly and positively correlated between colchicine-treated and control brains, suggesting that GAD65 expression in the soma can be used as a proxy for the overall amount of GAD65 in the cytoplasm. It is important to consider the possibility that the colchicine injections might have caused abnormal effects on not only axonal transport but also protein synthesis or metabolism, as colchicine is neurotoxic (Sutula et al., 1983). Neural excitation caused by colchicine might affect the GAD65 expression, because temporal lobe epilepsy caused by pilocarpine is reported to increase GAD65 expression (Esclapez and Houser, 1999). In additions, GABAergic neurons may have differing colchicine sensitivities based on their morphologies and localization patterns. In this study, we injected colchicine into the lateral ventricle of the brain. Layers near the lateral ventricle might have thus been vulnerable to drug effects. In future research, synaptic protein levels can be detected directly using super-resolution fluorescence microscopy. Complementary data should be obtained through several methods.

### GAD65-Expression in Subtypes Contributes Understanding of Pathological States

High GAD65 expression in NOS^+^ and NPY^+^ neurons has important implications. As noted in the introduction, GAD65 is associated with activity-dependent GABA release, whereas GAD67 is associated with spontaneous GABA release (Tian et al., 1999; Jinno and Kosaka, 2009). Our findings suggest that nNOS^+^ and NPY^+^ neurons are closely associated with activity-dependent GABA release. Among GABAergic subtypes, PV^+^ neurons are considered a pivotal subtype based on previous studies using subtype-specific GAD67-knockout or -knockdown models (Kuki et al., 2015; Lazarus et al., 2015). However, no studies to date have examined the functions of GAD65 in nNOS^+^ and NPY^+^ neurons. In humans, electroconvulsive therapy increases the seizure threshold (Sackeim, 1999) and GABA expression in the brain (Sanacora et al., 2003). According to our findings, GAD65 expression may increase in nNOS^+^ and NPY^+^ neurons, resulting in increased GABA expression and a higher seizure threshold. This study demonstrates the baseline level of GAD65 expression at the resting state of various GABAergic subtypes. In the future, the relationships between GABAergic subtypes and pathological states should be studied, as GABAergic subtypes play distinct roles in excitatory-inhibitory balance under normal and pathological conditions, including epileptic seizures and mental disorders.

## Data Availability Statement

The raw data supporting the conclusions of this article will be made available by the authors, without undue reservation.

## Ethics Statement

The animal study was reviewed and approved by the Institute for Animal Experimentation of Tohoku University (Permission number: 2019 IDOU-291-03).

## Author Contributions

YK and HM conceived and designed the experiments, wrote the manuscript, and contributed to the article and approved the submitted version. YK performed the experiments and analyzed the data. Both authors contributed to the article and approved the submitted version.

## Conflict of Interest

The authors declare that the research was conducted in the absence of any commercial or financial relationships that could be construed as a potential conflict of interest.

## Publisher’s Note

All claims expressed in this article are solely those of the authors and do not necessarily represent those of their affiliated organizations, or those of the publisher, the editors and the reviewers. Any product that may be evaluated in this article, or claim that may be made by its manufacturer, is not guaranteed or endorsed by the publisher.
